# Aerobic exercise training prevents obesity and insulin resistance independent of the renin angiotensin system modulation in the subcutaneous white adipose tissue

**DOI:** 10.1371/journal.pone.0215896

**Published:** 2019-04-25

**Authors:** Anna Laura V. Américo, Cynthia R. Muller, Bruno Vecchiatto, Luiz Felipe Martucci, Miriam H. Fonseca-Alaniz, Fabiana S. Evangelista

**Affiliations:** 1 Department of Experimental Pathophysiology, Faculty of Medicine, University of São Paulo, São Paulo, Brazil; 2 School of Arts, Science and Humanities, University of Sao Paulo, São Paulo, Brazil; 3 Heart Institute (InCor), Faculty of Medicine, University of Sao Paulo, São Paulo, Brazil; Max Delbruck Centrum fur Molekulare Medizin Berlin Buch, GERMANY

## Abstract

We investigate the effects of aerobic exercise training (AET) on the thermogenic response, substrate metabolism and renin angiotensin system (RAS) in the subcutaneous white adipose tissue (SC-WAT) of mice fed cafeteria diet (CAF). Male C57BL/6J mice were assigned into groups CHOW-SED (chow diet, sedentary; n = 10), CHOW-TR (chow diet, trained; n = 10), CAF-SED (CAF, sedentary; n = 10) and CAF-TR (CAF, trained; n = 10). AET consisted in running sessions of 60 min at 60% of maximal speed, five days per week for eight weeks. The CAF-SED group showed higher body weight and adiposity, glucose intolerance and insulin resistance (IR), while AET prevented such damages in CAF-TR group. AET reduced the p-AKT/t-AKT ratio and increased ATGL expression in CHOW-TR and CAF-TR groups and increased t-HSL and p-HSL/t-HSL ratio in CAF-TR. AET prevented adipocyte hypertrophy in CAF-TR group and increased UCP-1 protein expression only in CHOW-TR. Serum ACE2 increased in CHOW-TR and CAF-TR groups, and Ang (1–7) increased in the CHOW-TR group. In the SC-WAT, CAF-TR group increased the expression of AT1, AT2 and Mas receptors, whereas CHOW-TR increased Ang (1–7) and Ang (1–7)/Ang II ratio in SC-WAT. No changes were observed in ACE and Ang II. Positive correlations were observed between UCP-1 and kITT (r = 0.6), between UCP-1 and Ang (1–7) concentration (r = 0.6), and between UCP-1 and Ang (1–7)/Ang II ratio (r = 0.7). In conclusion, the AET prevented obesity and IR, reduced insulin signaling proteins and increased lipolysis signaling proteins in the SC-WAT. In addition, the CAF diet precludes the AET-induced thermogenic response and the partial modulation of the RAS suggests that the protective effect of AET against obesity and IR could not be associated with SC-WAT RAS.

## Introduction

Aerobic exercise training (AET) has been widely used for the prevention and treatment of obesity, insulin resistance (IR) and type 2 diabetes (T2D) because it is able to improve mitochondrial function and fatty acid oxidation [1, 2], reduces body weight and adiposity [3, 4], improves insulin sensitivity and glucose uptake in the skeletal muscle [5]. AET also prevents ectopic lipid accumulation [6, 7] and ameliorates inflammatory response in T2D patient [8].

The contribution of adipose tissue has provided insight into mechanisms underlying the positive metabolic effects of AET. In a previous study, our group demonstrated that AET prevented obesity and IR by improving lipolysis, reducing enzymes responsible for fatty acid esterification and activating enzymes that improve lipid oxidation instead of lipid storage in the white adipose tissue (WAT) [3]. The availability of substrate for storage in WAT is also determined by the thermogenic capacity of brown adipose tissue (BAT), which utilizes fatty acids and glucose for heat production via the mitochondrial uncoupling protein-1 (UCP-1) [9]. When activated, UCP1 performs proton pumping through the internal mitochondrial membrane, which uncouple oxidative respiration from ATP synthesis, increasing thermogenesis [10, 11]. Increases in the activity of BAT can raise whole-body energy expenditure and therefore can contribute for the prevention and treatment of obesity and T2D [12].

It has been reported that the differentiation of adipocytes with brown-like appearance and thermogenic phenotype in the WAT, called beige adipocyte, can contribute to increased energy expenditure [10,13–15]. Beige adipocytes have high activity of UCP-1 and therefore can be targeted to help counteract the development of metabolic diseases or to treat obese and diabetic individuals [10,14]. Different stimuli are potent to induce browning of WAT, such as AET, cold and beta-adrenergic drugs [16–18]. With respect of AET, Wu et al. [19] showed that AET induced browning of the subcutaneous WAT (SC-WAT) in rats, and despite reducing the thermogenic capacity of the BAT, the whole-body energy expenditure still increased in the trained rats due to the browning of SC-WAT.

The WAT plays an important role in producing specific endocrine proteins, pro-inflammatory cytokines, and metabolites that are involved in the control of energy metabolism, body weight, glycemic homeostasis and lipid metabolism [20, 21]. It was showed that the WAT expresses the components of the renin angiotensin system (RAS), including angiotensin converting enzyme (ACE), angiotensin II (Ang II) and AT1 receptor (ACE/Ang II/AT1 axis), which is hyperactivated in metabolic diseases [22–24]. Ang II induces lipogenesis and reduces lipolysis in the WAT which is related to obesity, IR and inflammation [25–31].

A counter-regulatory RAS axis consists of ACE2, an ACE homologue enzyme, which converts Ang II into angiotensin (1–7) (Ang (1–7)), which in turn binds to the Mas receptor (ACE2/Ang (1–7)/Mas). This axis is also expressed in the WAT [32–36], however when activated it induces metabolic actions such as increase in lipolysis [37], reduction in body weight and improve in lipid profile [38–40], attenuation in the metabolic syndrome, increase in glucose uptake and reduction in oxidative stress [41, 42]. Because of these protective responses, the ACE2/Ang (1–7)/Mas axis counteracts the deleterious effect of ACE/Ang II/AT1 axis and have been investigated as a target for reducing obesity and DM2 [29, 36, 43].

Some effects of AET on RAS have been shown in the literature such as systemic and tissue ACE/Ang II/AT1 axis reduction [44–48], a shift in the RAS towards the ACE2/Ang (1–7)/Mas axis in the skeletal muscle [49] and in the liver of trained rats fed a fructose overload [30]. However, the effect of AET on the WAT RAS and the repercussions for the prevention of obesity and IR still needs investigation. Therefore, the aim of this study was to investigate the effects of AET on the thermogenic response, substrate metabolism and RAS in the SC-WAT of mice fed cafeteria diet.

## Materials and methods

### Animals

Eight-week-old male C57BL/6J mice were assigned in groups CHOW-SED (chow diet, sedentary; n = 10), CHOW-TR (chow diet, trained; n = 10), CAF-SED (cafeteria diet, sedentary; n = 10) and CAF-TR (cafeteria diet, trained; n = 10). Animals were maintained under the same housing conditions (12-h light/12-h dark cycle, temperature 22 ± 2°C) with free access to tap water and food *ad libitum*. All procedures were approved by the ethics committee of the Faculty of Medicine of University of Sao Paulo (# 002/15).

### Diets and aerobic exercise training

The standard chow diet contained 4% of kilocalories from fat, 55% from carbohydrate and 22% from proteins (Nuvilab, Paraná, Brazil). The cafeteria diet contained 18.8% of kilocalories from fat, 55% from carbohydrate and 14.8% from proteins [3]. Diet and AET were started simultaneously. CHOW-TR and CAF-TR animals were submitted to AET as described by [50]. Animals were trained during the dark cycle (i.e., during their active period) on a motorized treadmill for 1 h/day at 60% of maximal velocity achieved in the running capacity test, five times per week for eight weeks. AET intensity was progressively increased and adjusted after the running capacity test done in the fourth week. To minimize the influence of the treadmill stress, sedentary mice were placed on the treadmill for 5 min twice weekly at 0.3 km/h during the experimental protocol.

### Running capacity test

Running capacity was assessed before, in the fourth and eighth weeks of AET using a progressive test without inclination on a treadmill as described by [50]. The initial speed was 0.4 km/h and the speed was increased by 0.2 km/h every three minutes until exhaustion of the animal, which was characterized by the impossibility of maintaining the standard rate.

### Indirect calorimetry

In the eighth week, the animals were acclimatized in the Oxylet Calorimetry System (Panlab, Barcelona, Spain) and the measurements were done during resting. Firstly, the animals were submitted to fasting (2-h) and then the volumes of oxygen consumption (VO_2_) and carbon dioxide production (VCO_2_) were measured during 45 min of resting. The non-protein respiratory exchange ratio (RER), a measurement of metabolic substrate preference, was calculated as the molar ratio of VCO_2_ to VO_2_. Energy expenditure (EE) was calculated using the formula: EE = [3.815 + (1.232 x RER)] x VO_2_ x 1.44. Carbohydrate (CHO) and lipids (LIP) oxidation rates were calculated as described by [51] CHO = (4.55 x VO_2_)—(3.21 x VCO_2_) and LIP = (1.67 x VO_2_)—(1.67 x VCO_2_). The EE was expressed as kcal. kg^-1^.min^-1^ and substrate oxidation was expressed as mg.kg^-1^.min^-1^.

### Body weight control and food intake

Throughout the experimental protocol, body weight was measured weekly in a digital balance (Gehaka / model BG4001, São Paulo, Brazil) in the same day and time. In addition to the evolution of body weight, the body weight gain was calculated by the difference between the final body weight (week 8) and the initial body weight (week 0). The 24-h food intake was determined weekly throughout the study in mice that were housed at three or four animals per cage.

### Glucose tolerance test (GTT) and insulin tolerance test (ITT)

GTT and ITT were performed after the 8-week of AET protocol. Both experiments were performed in awake animals after an 8-h fast. The glucose load (2 g/kg body weight) was injected as a bolus intraperitoneally, and the blood glucose levels were determined in caudal blood sampled at 0, 15, 30, 60, 90 and 120 min after glucose infusion. The glucose concentration was determined using a glucometer (AccuChek Advantage Roche Diagnostics).

After 72 h of GTT test, a similar procedure was performed for ITT. The insulin load (0.75 U/kg body weight) was injected as a bolus intraperitoneally, and the blood glucose levels were determined in caudal blood samples collected at 0, 5, 10, 15, 20, 25 and 30 min after injection. The values obtained between 5 and 30 min were used to calculate the rate constant for the disappearance of plasma glucose (kITT) according to the method proposed by [52].

### Tissue and blood collection

Forty-eight hours after the end of the last training session, the animals were killed with an intraperitoneal injection of pentobarbital sodium (4 mg/100 g body weight). Subcutaneous (inguinal) and visceral (periepididymal and retroperitoneal) WAT fat pads (SC-WAT, PE-WAT and RP-WAT, respectively) and interscapular brown adipose tissue (iBAT) were harvested, weighed and only SC-WAT was stored at 80° C. Blood was collected and centrifuged at 4° C, 12000 rpm for 10 minutes, and thereafter the serum was collected and stored in a freezer at -80 ° C.

### Histological analysis

Adipocyte morphology was measured in paraffin sections of SC-WAT fat pad (5μm) stained with hematoxylin and eosin (Sigma). Digital images from 50 adipocytes per animal were obtained using a light microscope (Leica) at 400x magnification. After digitalization, adipocyte diameter and area were traced and calculated using a computerized morphometric analysis system (Image Pro-Plus 4.1; Media Cybernetics, Silver Spring, MD, USA). The analysis was done by a single observer (Américo ALV) blinded to mice identities.

### Immunohistochemistry analysis

After deparaffinization in xylene and hydration in graded alcohols, antigen retrieval was performed in 5 μm thick SC-WAT slices by heating the slides in 0.01mol/L citrate buffer, pH 6.0. The endogenous peroxidase blockade present in the red blood cells was made with hydrogen peroxide 10v. Protein blocking was done with PBS solution pH 7.6 with 0.5% BSA and 0.5% casein (Spring). Primary antibody (UCP1, 1:1000; Abcam ab23841) was applied overnight at 4°C and subsequently incubated with the anti-rabbit N-Histofine polymer antibody to mouse tissue (Nichirei Bioscience Inc.). All slides were developed in DAB (Sigma-Aldrich Chemie, Steinheim, Germany), and counterstained in Harris hematoxylin (Merck, Darmstadt, Germany). Ten photos per slide were taken at 400X magnification using the Leica QWin Plus V3 program and were analyzed with the Image Pro Plus program by a single observer (Américo ALV) blinded to mice identities. The results are presented as percentage of positive cells per tissue area.

### mRNA quantification

Total RNA was extracted from the SC-WAT using TRIzol reagent according to the product technical specifications (Invitrogen Life Technologies, USA). The integrity of the RNA was evaluated by means of 0.8% agarose gel electrophoresis and by spectrophotometer quantification at 260 and 280nm. Only samples whose 260 / 280nm ratio was greater than 1.8 were used. The cDNA was synthesized using Superscript III RNase H-RT (Invitrogen Life Technologies, USA) at 42° C for 50 min. The primers used for amplification of the studied genes were: UCP-1(Foward:5’-CGATGTCCATGTACACCAAGGA-3’; Reverse:5’-TCGCAGAAAAGAAGCCACAA-3’), UCP-2(Foward:5’-GGCCTCTGGAAAGGGACTTC-3’; Reverse:5’-ACCAGCTCAGCACAGTTGACA-3’) and CIDEA (Foward:5’-TGCTCTTCTGTATCGCCCAGT-3’; Reverse:5’-GCCGTGTTAAGGAATCTGCTG-3’). The expression of 18S (Foward: 5’-TCGGCGTCCCCCAACTTCTTA-3’; Reverse: 5’- GGTAGTAGCGACGGGCGGTGT-3’) was measured as an internal control for sample variation in the reverse transcriptase (RT) reaction.

Gene expression evaluation was performed by real-time PCR (RT-PCR) and the amplifications were evaluated through the ABI Prism 7700 Sequence Detection System detection system (Applied Biosystems, Foster City, CA) in 384 well plates using Power SYBR Green Master Mix reagent (Applied Biosystems—USA). All samples were analyzed in duplicate. All target and reference genes were evaluated on the same plate under the same conditions. The comparative threshold cycle (CT) method was used for data analysis. CT indicates the number of the fractional cycle in which the amount of amplified target reaches a fixed threshold and ΔCT is the difference in the threshold cycle for the target and reference genes. The levels of gene expression are given by 2-ΔΔCt, where ΔΔCT is the ΔCT value subtracted from the ΔCT of the control group.

### Western blot determination

Samples of frozen SC-WAT were homogenized in lysis buffer with 1 M HEPES, 2 M NaCl, 20% SDS, 0.5 M EDTA, 100mM Benzamidina (pH = 7.4), protease and phosphatase inhibitor cocktail EDTA-free (Thermo Scientific) at the concentration of 10 μl/ml. Samples were centrifuged for 15 min at 15.000 rpm at 4°C. Protein concentrations of the homogenates were measured by the BCA method with a protein assay kit (PIERCE Biotechnology, Rockford, IL, USA) using bovine serum albumin as a standard. Aliquots (60 μg) of protein were subjected to SDS-PAGE. The membranes were incubated overnight at 4°C with the following primary antibodies: p- AKT (1:1000), t-AKT (1:1000), GLUT-4 (1:1000), AT2 (1:1000), MAS (1.25:1000) (Abcam, Cambridge, USA), p-HSL (1:1000), t-HSL (1:1000), Perilipin (1:1000), ATGL (1:1000), AT1 (1:1000), (Cell Signaling, Beverly, MA) and beta-actin (Abcam, Cambridge, USA). The signal on the membrane was detected via the peroxidase reaction in the ECL solution using an Image Quant LAS 4000 mini system (GE Healthcare Life Sciences). Band intensities were quantified based on optical densitometry measurements using the Image J program (version 1.43 for Windows).

### Enzyme assay

ACE and ACE2 activities were evaluated in SC-WAT and serum using fluorescent peptides [53–54]. For ACE assay, the samples were incubated with a solution of Abz-FRK(Dnp)P-OH (Abz = ortho-aminobenzoic acid; Dnp = dinitrophenyl) (15 μM) in 0.1 M Tris-HCl buffer containing 50 mM NaCl and 10 mM ZnCl2 (pH 7.0). For ACE2 assay, Abz-APK peptide (Dnp)-OH in 0.2 M Tris-HCl buffer, 200 mM NaCl, pH 7.5, was used. The enzymatic activity was determined by measuring the fluorescence for 10 minutes (one reading per minute). Protein concentrations of the homogenates were measured by the BCA method with a protein assay kit (PIERCE Biotechnology, Rockford, IL, USA) using bovine serum albumin as a standard. The activities were expressed in UF/μg of tissue protein or UF/mL in serum.

### Peptide assay

The concentration of Ang II and Ang (1–7) were evaluated in SC-WAT fat pad and Ang II in the serum by ELISA using commercial kits (Biomatik Corp., Cambridge, Ontario) according to the manufacturer’s instructions.

### Statistical analyses

All values are expressed as mean ± SE. The normality distribution of each variable was tested by Shapiro-Wilk. When the supposed normality was not satisfied, we performed Kruskal Wallis test. Data with normal distribution were analyzed with two-way analyses of variance (ANOVA). The Tuckey *post hoc* test was used to determine differences between means when a significant change was observed using ANOVA. A p value equal to or less than 0.05 was statistically significant (GraphPad Prism, v.7.0).

## Results

### Physical performance and glucose metabolism

Before AET, no differences in running capacity test were observed among groups (CHOW-SED = 46.1 ± 1.3 cm/s; CHOW-TR = 48.3 ± 1.1 cm/s; CAF-SED = 48.3 ± 1.9 cm/s; CAF-TR = 47.2 ± 0.8 cm/s; n = 10/group). However, after 8 weeks of AET the physical performance improved based on the increase in running velocity (F = 18.93, P = 0.0001) of CHOW-TR (64.44 ± 11.42 cm/s) compared with sedentary groups (CHOW-SED = 52.33 ± 10.46 cm/s, P = 0.0202 and CAF-SED = 49 ± 6.09 cm/s, P = 0.0048) and CAF-TR (62.25 ± 2.31 cm/s, P = 0.0224) compared with CAF-SED.

In Table 1, it was showed the results of resting indirect calorimetry. There was no difference in the values of VO_2_, VCO_2_, RER and EE among groups. Also, AET and cafeteria diet did not change CHO and LIP oxidation rates during rest.

**Table 1:** Metabolic parameters measured during the rest.

	CHOW-SED (n = 10)	CHOW-TR (n = 9)	CAF-SED (n = 8)	CAF-TR (n = 8)
**VO**_**2**_ **(ml/min/kg)**	48.68 ± 1.54	39.78 ± 2.43	49.56 ± 13.2	41.54 ± 4.17
**VCO**_**2**_ **(mL/min/kg)**	34.03 ± 1.38	27.17 ± 2.14	33.33 ± 9.81	28.32 ± 3.10
**RER**	0.70 ± 0.01	0.68 ± 0.2	0.67 ± 0.03	0.68 ± 0.01
**EE (kcal. kg**^**-1**^**.min**^**-1**^**)**	315.79 ± 17.5	266.74 ± 16.8	323.33 ± 57.2	278.46 ± 18.1
**CHOox (mg. kg**^**-1**^**.min**^**-1**^**)**	112.23 ± 3.8	93.79 ± 5.94	118.50 ± 29.29	98.09 ± 9.26
**LIPox (mg.kg**^**-1**^**.min**^**-1**^**)**	24.46 ± 1.26	21.06 ± 1.9	27.1 ± 6.32	22.07 ± 2.0

Data are presented as mean ± SE. VO_2_ = oxygen consumption; VCO_2_ = carbon dioxide production; RER = respiratory exchange ratio; EE = energy expenditure; CHOox = carbohydrate oxidation; LIPox = lipid oxidation. The results were compared by the two-way ANOVA.

**Fig 1:**
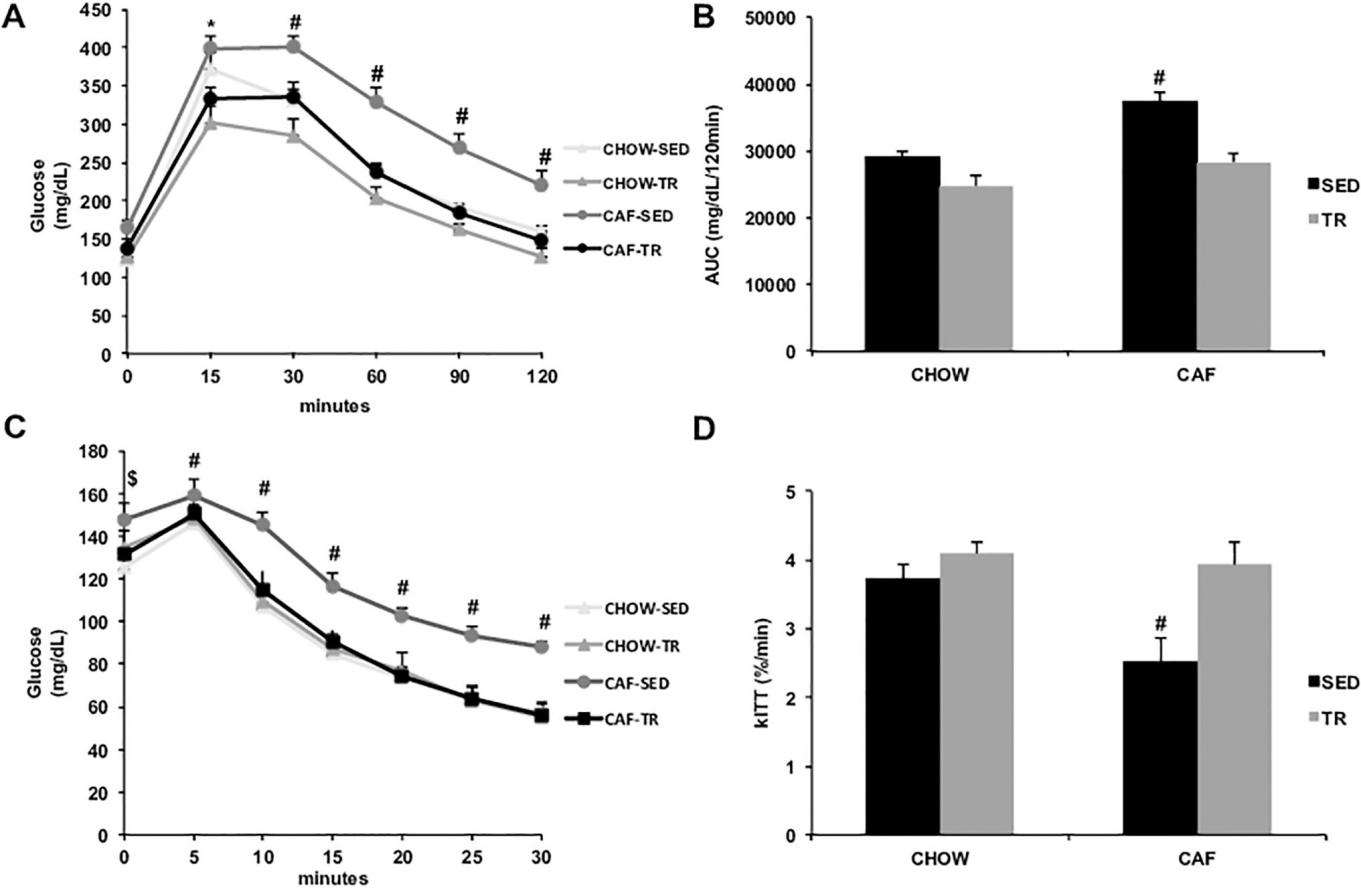
Glucose tolerance test (A), area under the curve (B), insulin tolerance test (C) and kITT (D). CHOW-SED (n = 10), CHOW-TR (n = 10), CAF-SED (n = 10) and CAF-TR (n = 10). AUC = area under the curve; kITT = glucose disappearance constant rate. Error bars indicate the SE. p≤ 0.05 *CHOW-SED vs. CHOW-TR, ^#^CAF-SED vs. CHOW-SED, CHOW-TR and CAF-TR, ^$^CAF-SED vs CHOW-SED. The results were compared by the two-way ANOVA.

In the GTT, glucose clearance was lower in CAF-SED animals compared with other groups (Fig 1A) (Interaction F = 2.394, P = 0.0039; Time F = 284.9, P < 0.0001; Group F = 15.42, P < 0.0001), which was confirmed by the higher area under the glycemic curve (AUC) in CAF-SED group (Fig 1B) (Diet F = 21.4, P < 0.0001; Exercise F = 26.94, P < 0.0001). These data confirm the glucose intolerance presented by CAF-SED and that AET prevented this response in the CAF-TR group. In the ITT, CAF-SED group showed a decrease in the insulin-stimulated glucose decay, remaining with the glucose level higher than the other groups at the end of the test (Fig 1C) (Interaction F = 2.124, P = 0.0066; Time F = 399.3, P < 0.0001; Group F = 5.426, P = 0.0041). The same can be evidenced in the kITT (Fig 1D) (Diet F = 6.223, P = 0.0181; Exercise F = 10.72, P = 0.0026), revealing that CAF-SED developed resistance to insulin and that AET prevented this response in CAF-TR group.

### Body weight, food intake and adiposity

**Fig 2:**
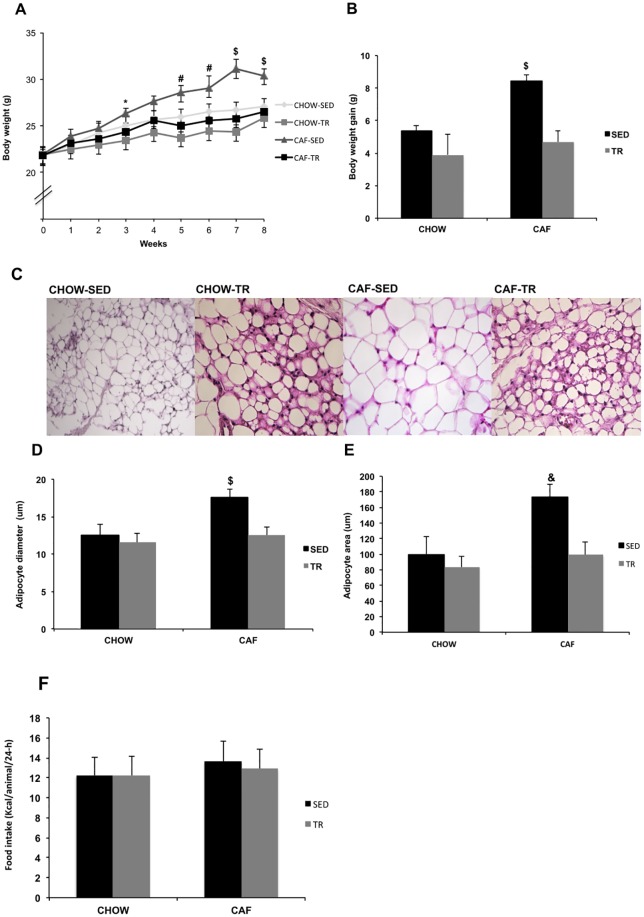
Body weight evolution (A), body weight gain (B), representative photomicrographs of histological sections of SC-WAT stained with hematoxylin and eosin to visualize the morphology of adipocytes at 400X magnification (C), adipocyte diameter (D), adipocyte area (E) and food intake (F). CHOW-SED (n = 10), CHOW-TR (n = 10), CAF-SED (n = 10), CAF-TR (n = 10). Error bars indicate the SE. p≤ 0.05 *CAF-SED vs. CHOW-TR, ^#^CAF-SED vs. CHOW-TR and CAF-TR, ^$^CAF-SED vs. CHOW-SED, CHOW-TR and CAF-TR, ^&^CAF-SED vs. CHOW-SED, CHOW-TR. The results of body weight evolution, body gain and food intake were compared by the two-way ANOVA; The result of adipocyte diameter and area were compared by the Kruskal Wallis test.

The initial body weight did not differ among groups, however the CAF-SED group presented greater body weight compared with CHOW-TR group at 3^rd^ week (Fig 2A). From week 5, CAF-SED had higher body weight compared with both trained groups CHOW-TR and CAF-TR. In the 7^th^ week, it was found increase in the body weight of CAF-SED compared with the other groups (Fig 2A) (Interaction F = 4.621, P < 0.0001; Time F = 79.61, P < 0.0001; Group F = 4.727, P = 0.007). Moreover, the CAF-SED group showed higher body weight gain compared with the other three experimental groups (F = 6.508, P = 0.0151) and the AET was able to prevent increased body weight gain in CAF-TR group (Fig 2B). The weight of SC-WAT was higher in CAF-SED compared with CHOW-SED (Mean rank diff = 16.67, P = 0.05) and CHOW-TR (Mean rank diff = 20.33, P = 0.007) (Table 2). Furthermore, the weight of PE-WAT was higher in CAF-SED compared with CHOW-SED (Mean rank diff = 17.43, P = 0.03), CHOW-TR (Mean rank diff = 24.9, P = 0.0004) and CAF-TR (Mean rank diff = 23.07, P = 0.0014) groups (Table 2). The RP-WAT and iBAT weight were not different among groups (Table 2).

**Table 2:** Adipose tissue weight.

Fat Pad (mg/g)	CHOW-SED (n = 10)	CHOW-TR (n = 10)	CAF-SED (n = 10)	CAF-TR (n = 10)
**SC-WAT**	0.011 ± 0.001	0.01 ± 0.001	0.029 ± 0.007*	0.021 ± 0.004
**PE-WAT**	0.007 ± 0.001	0.005 ± 0.001	0.02 ± 0.005^#^	0.015 ± 0.002
**RP-WAT**	0.003 ± 0.0004	0.007 ± 0.004	0.011 ± 0.002	0.007 ± 0.002
**iBAT**	0.003 ± 0.0003	0.004 ± 0.001	0.004 ± 0.001	0.004 ± 0.001

Data are presented as mean ± SE.

* p ≤ 0.05 vs. CHOW-SED and CHOW-TR.

^#^p ≤ 0.05 vs. CHOW-SED, CHOW-TR and CAF-TR.

The results were compared by the Kruskal Wallis test.

Histological analysis in the SC-WAT revealed that CAF-SED animals had an increase in adipocyte diameter compared with CHOW-SED (Mean rank diff = 14.41, P = 0.035), CHOW-TR (Mean rank diff = 15.41, P = 0.019) and CAF-TR groups (Mean rank diff = -14.1, P = 0.042), and the adipocyte area was higher in CAF-SED compared with CHOW-SED (Mean rank diff = 14.4, P = 0.035) and CHOW-TR (Mean rank diff = 15.7, P = 0.016) and showed a tendency compared with CAF-TR group (Mean rank diff = -13.5, P = 0.058) (Fig 2C, 2D and 2E). These findings suggest that cafeteria diet induced adipocyte hypertrophy and that AET was able to prevent this response in CAF-TR group. These responses were independent of food consumption changes because no differences were observed in 24-h food intake among groups (Fig 2F).

### Thermogenic brown features in the SC-WAT

The UCP-1 gene expression showed a non-significant increase of 116% (P = 0.06) in the CHOW-TR group compared to CHOW-SED group. It was also observed a non-significant reduction in UCP-1 gene expression (87%, P = 0.08) in CAF-SED compared to CHOW-SED group (Table 3). The cafeteria diet reduced UCP-2 gene expression in CAF-SED group compared with CHOW-SED and CHOW-TR (F = 13.02; P = 0.0026). Also, cafeteria diet reduced CIDEA gene expression in both CAF-SED and CAF-TR groups compared with the CHOW-TR group (Diet F = 8.758, P = 0.0097; Exercise F = 13.24, P = 0.0024). (Table 3).

**Table 3:** mRNA expression in SC-WAT.

Gene	CHOW-SED (n = 5)	CHOW-TR (n = 5)	CAF-SED (n = 5)	CAF-TR (n = 5)
**UCP-1**	1.0 ± 0.19	2.16 ± 0.91	0.13 ± 0.04	1.26 ± 0.9
**UCP-2**	1.0 ± 0.04	1.02 ± 0.08	0.47 ± 0.09*	0.71 ± 0.2
**CIDEA**	1.0 ± 0.24	2.1 ± 0.21	0.23 ± 0.06^#^	0.81 ± 0.42 ^#^

Data presented as mean ± SE.

*p ≤ 0.05 vs. CHOW-SED and CHOW-TR,

^#^p ≤ 0.05 vs. CHOW-TR.

The results were compared by the two-way ANOVA.

**Fig 3:**
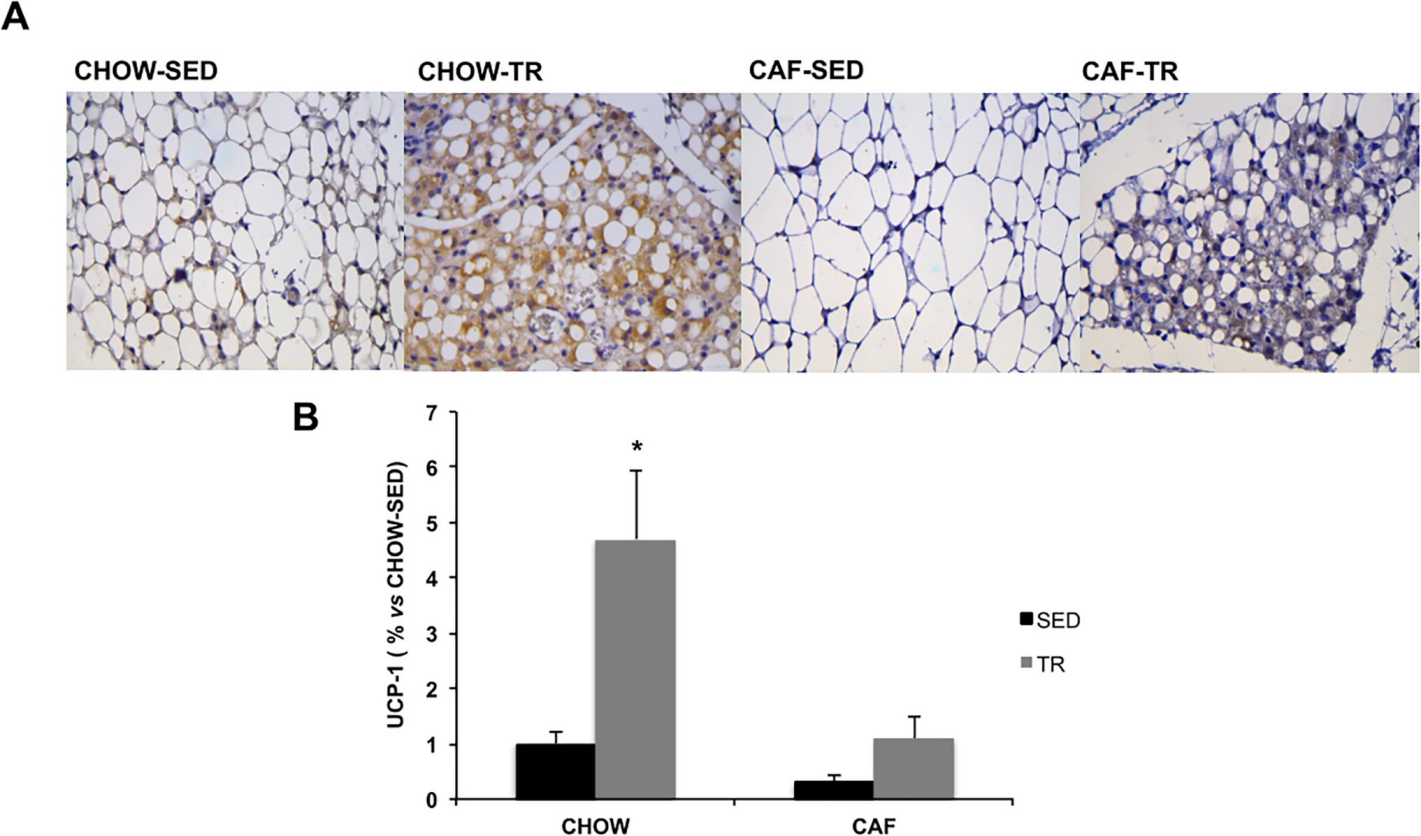
Representative photomicrographs of histological sections of SC-WAT with UCP-1 expression (positive marking in brown) at 400X magnification (A) and content of UCP-1 in SC-WAT (B). CHOW-SED (n = 5), CHOW-TR (n = 5), CAF-SED (n = 5) and CAF-TR (n = 5). * p <0.05 vs CHOW-SED, CAF-SED, CAF-TR. The results were compared by the two-way ANOVA.

The UCP-1 content was measured to identify AET-induced browning effects in the SC-WAT. As showed in the Fig 3A and 3B, AET increased UCP-1 expression in CHOW-TR compared with the other groups (Interaction F = 10.5, P = 0.0064; Exercise F = 18.91, P = 0.0008; Diet F = 21.49, P = 0.0005). This finding indicates that AET induced the appearance of brown-like adipocyte in SC-WAT and that cafeteria diet attenuates this effect in CAF-TR group.

### Markers of tissue metabolism

**Fig 4:**
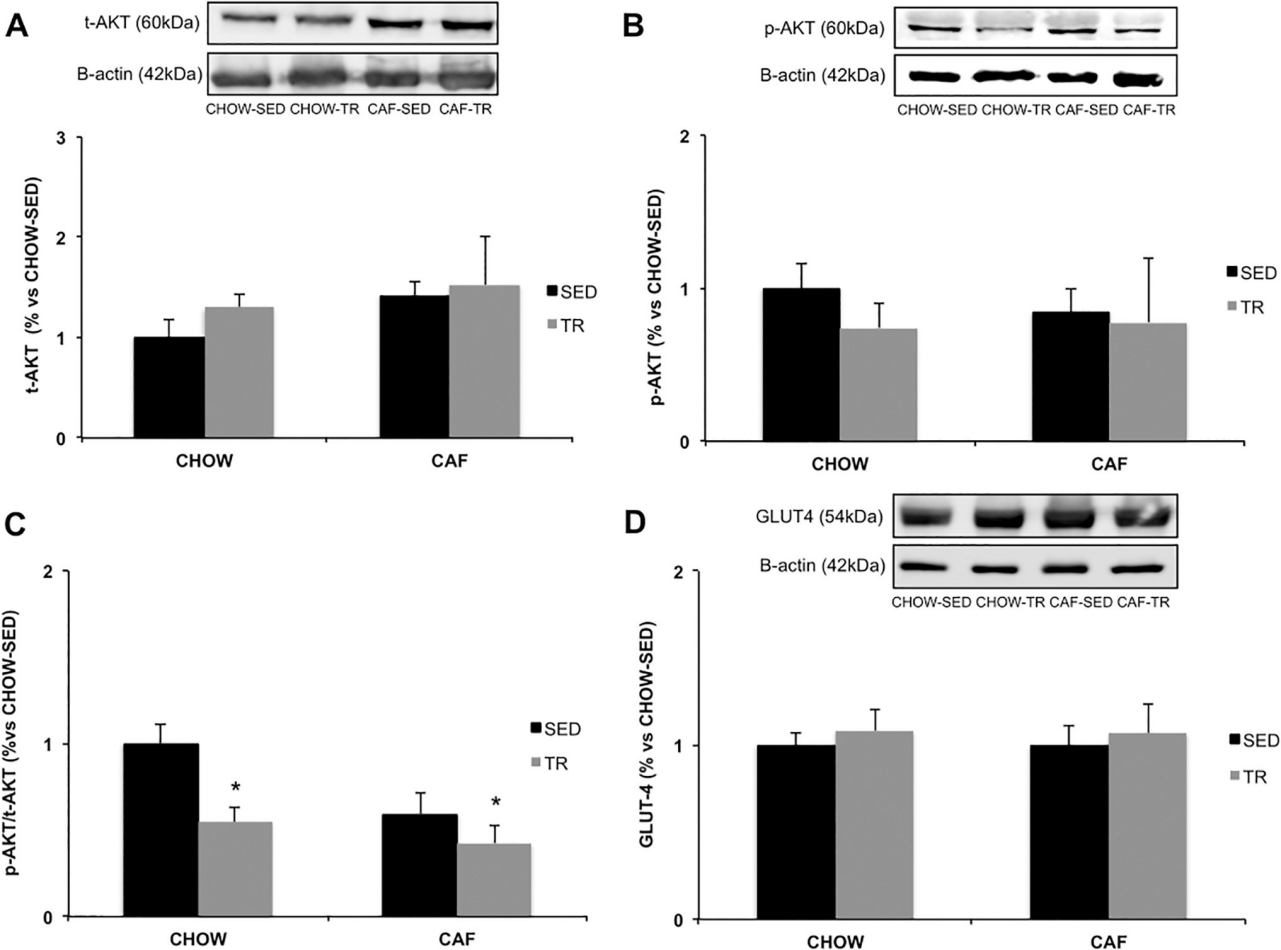
Protein expression of total AKT (t-AKT) (n = 6/group) (A), phosphorylated AKT (p-AKT) (n = 4/group) (B), p-AKT / t-AKT ratio (n = 4/group) (C) and glucose transporter Glut-4 (n = 5/group) (D) in SC-WAT. * p <0.05 vs CHOW-SED. The results were compared by the two-way ANOVA.

**Fig 5:**
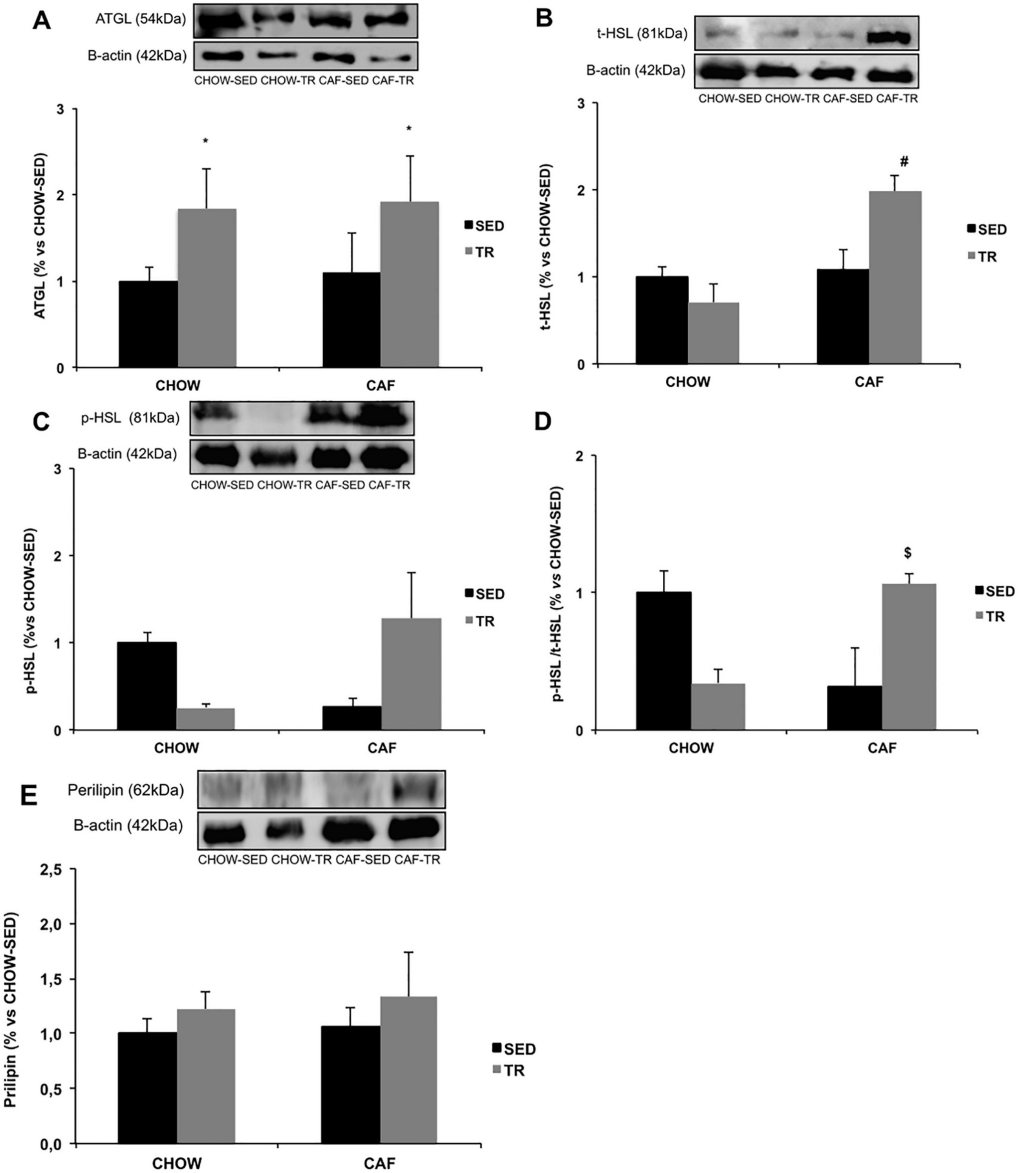
Protein expression of ATGL (n = 6/group) (A), total HSL (t-HSL) (n = 5/group) (A), phosphorylated HSL (p-HSL) (n = 4/group) (B), p-HSL / t-HSL ratio (n = 4/group) (C), perilipin (n = 6/group) (D) in SC-WAT. *p <0.05 vs CHOW-SED and CAF-SED; ^#^p <0.05 vs CHOW-TR; ^$^p <0.05 vs CHOW-TR and CAF-SED. The results were compared by the two-way ANOVA.

The content of proteins involved in glucose and lipid metabolism were measured in the SC-WAT. The expressions of t-AKT (Fig 4A) and p-AKT proteins (Fig 4B) were not different, but p-AKT/t-AKT ratio was lower in both trained groups compared with CHOW-SED (Exercise F = 8.239, P = 0.015; Diet F = 5.913, P = 0.033) (Fig 4C). There was no difference in GLUT-4 glucose transporter protein expression (Fig 4D). In the CHOW-TR and CAF-TR groups, the expression of ATGL protein increased when compared with both sedentary groups CHOW-SED and CAF-SED (Exercise F = 5.336, P = 0.039) (Fig 5A). CAF-TR group showed higher t-HSL protein expression in SC-WAT compared with CHOW-TR group (Interaction F = 7.704, P = 0.014; Diet F = 6.981, P = 0.019) (Fig 5B). No difference was observed in p-HSL protein expression (Fig 5C), however the ratio of p-HSL/t-HSL was higher in CAF-TR compared with CHOW-TR and CAF-SED groups (Interaction F = 23.64, P = 0.0028) (Fig 5D). It was not found any difference in the perilipin protein expression among the studied groups (Fig 5E).

### RAS content

Table 4 shows data from enzymes activities and peptide concentrations. Serum ACE activity was not different among groups. However, AET increased serum ACE2 activity in CHOW-TR group compared with CHOW-SED and CAF-SED groups (Exercise F = 5.336, P = 0.001). Ang (1–7) circulated levels was higher in the CHOW-TR and CAF-TR compared with sedentary groups (Exercise F = 8.324, P = 0.012). In SC-WAT, it was not found differences in the ACE activity and Ang II content among groups, however we observed an increase in the ACE2 activity of CAF-SED and CAF-TR compared with CHOW-SED and CHOW-TR (Diet F = 4.675, P = 0.039). AET induced an increase in the Ang (1–7) concentration in CHOW-TR compared with the other groups (Interaction F = 5.103, P = 0.039; Exercise F = 4.692, P = 0.004), which reveals that cafeteria diet precluded this response in CAF-TR group (Table 4).

**Table 4:** Enzyme activity and peptide content in serum and SC-WAT.

	CHOW-SED	CHOW-TR	CAF-SED	CAF-TR
***Serum***				
ACE (UF/mL; n = 5)	1597.7 ± 73.7	1586.8 ± 84.8	1515.8 ± 94.7	1923.4 ± 123.1
ACE2 (UF/mL; n = 5)	632628±122675	1270349±74399*	564363±144317	879031±102690
Ang (1–7) (pg/mL/μg; n = 5)	3.7 ± 0.6	7.1 ± 1.9*	4.0 ± 0.4	8.1 ± 1.1*
***SC-WAT***				
ACE (UF/μg; n = 10)	5307 ± 280	4044 ± 322	5782 ± 679	5678 ± 457
Ang II (pg/mL/μg; n = 5)	11733 ± 4407	19024 ± 2164	18218 ± 4859	19454 ± 1727
ACE2 (UF/μg; n = 10)	1531 ± 108	1577 ± 144	2072 ± 261^#^	1909 ± 225^#^
Ang (1–7) (pg/mL/μg; n = 5)	3322 ± 1446	12445 ± 3359^$^	3635 ± 1251	3444 ± 949

Data presented as mean ± SE.

*p ≤ 0.05 vs. CHOW-SED and CAF-SED,

^#^p ≤ 0.05 vs. CHOW-SED and CHOW-TR,

^$^p ≤ 0.05 vs. CHOW-SED, CAF-SED and CAF-TR.

The results were compared by the two-way ANOVA.

**Fig 6:**
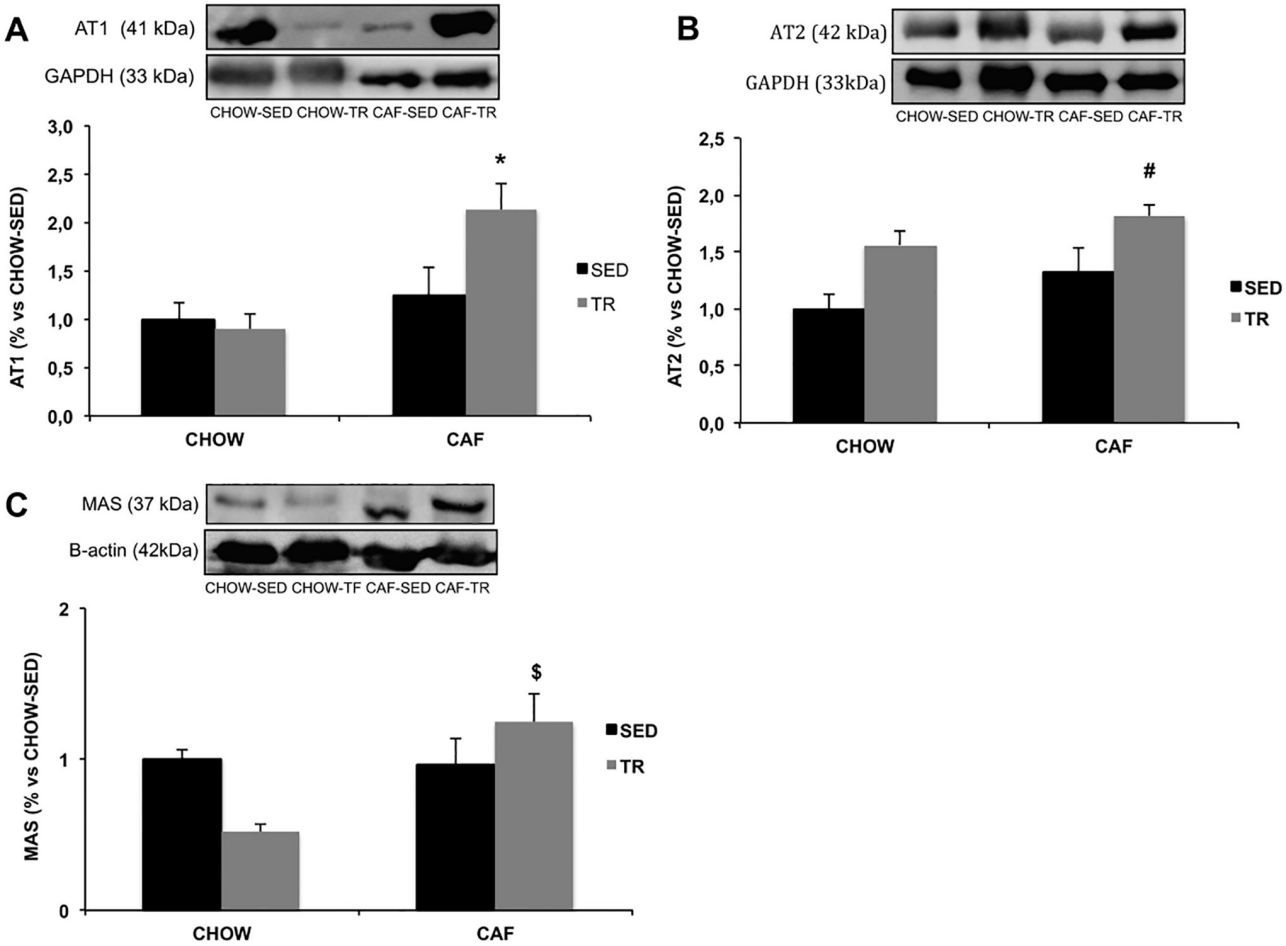
Protein expression of AT1 (n = 3/group) (A), AT2 (n = 8/group) (B) and Mas (n = 4/group) (C) receptors in SC-WAT. *p <0.05 vs CHOW-SED and CHOW-TR; ^#^p <0.05 vs CHOW-SED and CAF-SED; ^$^p <0.05 vs CHOW-TR. The results of AT1 and AT2 were compared by the two-way ANOVA; The result of Mas was compared by the Kruskal Wallis test.

The expression of AT1 receptor was higher in CAF-TR compared with CHOW-SED and CHOW-TR groups (Diet F = 9.018, P = 0.017) (Fig 6A). Moreover, AT2 receptor expression was higher in CAF-TR group compared with CHOW-SED and CAF-SED (Diet F = 5.394, P = 0.0037; Exercise F = 10.35, P = 0.029) (Fig 6B). We also observed 50% of increase in the AT2 expression of CHOW-TR compared with CHOW-SED (P = 0.06) (Fig 6B). For the protein quantification of Mas receptor (Mean rank diff = 8.5, P = 0.046) (Fig 6C), it was observed an increase in CAF-TR group compared with CHOW-TR.

**Fig 7:**
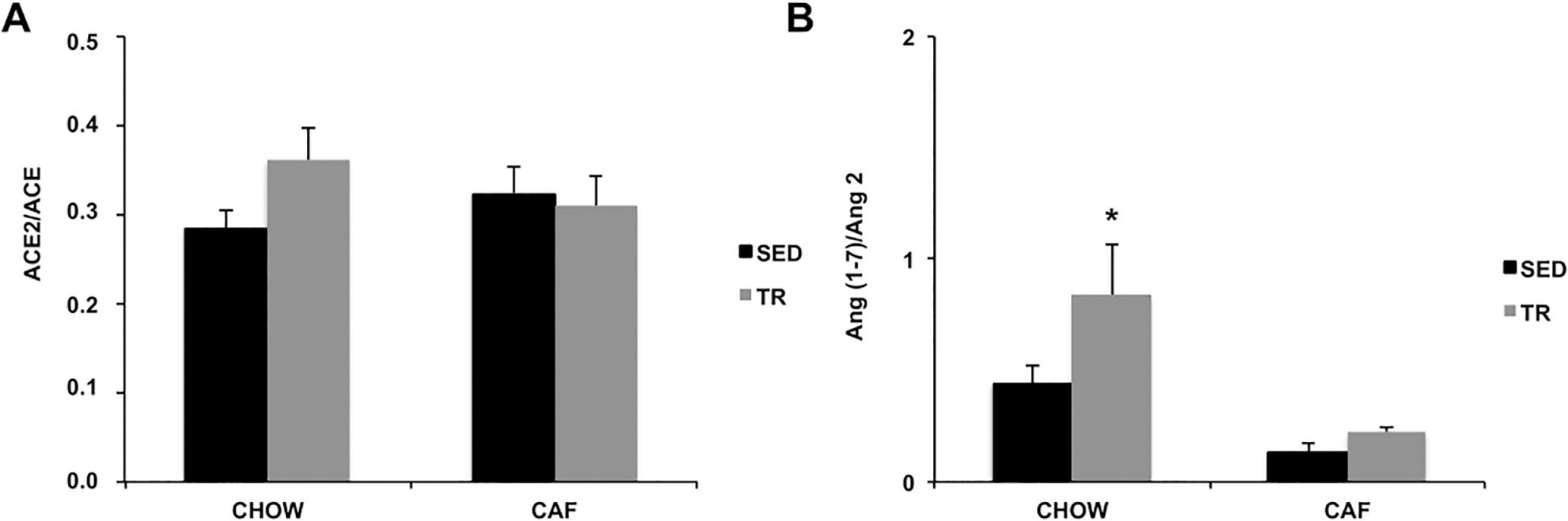
ACE2/ACE activity (n = 10/group) (A) and Ang (1–7)/Ang II ratio (n = 5/group) (B) in SC-WAT. *p <0.05 vs CAF-SED and CAF-TR. The results were compared by the two-way ANOVA.

The calculation of ACE2/ACE activities ration in the SC-WAT was not different among groups (Fig 7A), however the Ang (1–7)/Ang II ration was higher in CHOW-TR compared with CAF-SED and CAF-TR (Diet F = 13.73, P = 0.0026) (Fig 7B).

### Correlation analysis

**Fig 8:**
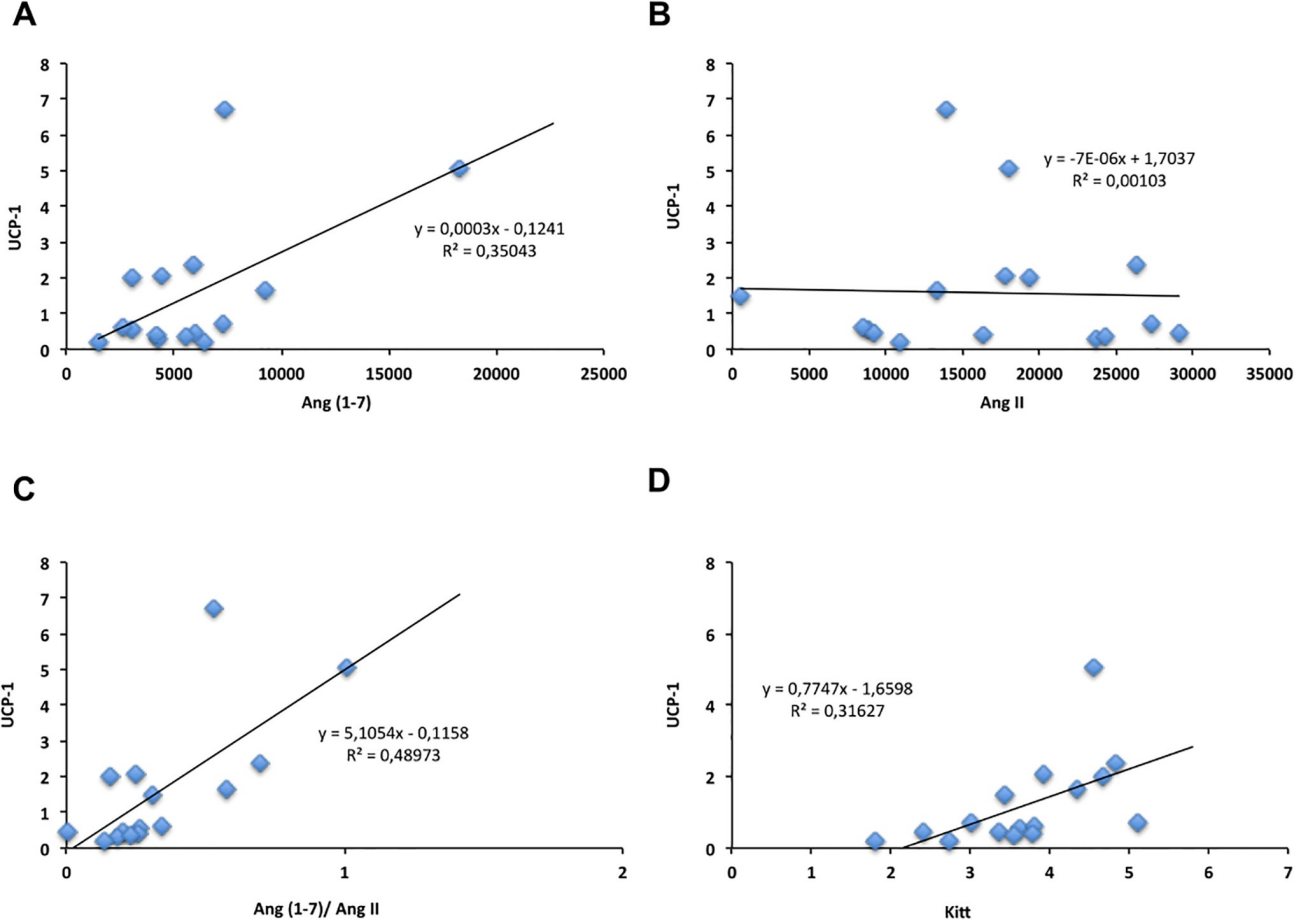
Correlation of UCP-1 and Ang (1–7) (A), UCP-1 and Ang II (B), UCP-1 and Ang (1–7)/AngII ratio (C), UCP-1 and Kitt (D). CHOW-SED (n = 5), CHOW-TR (n = 5), CAF-SED (n = 5) and CAF-TR (n = 5).

Positive correlation was found between UCP-1 and Kitt (r = 0.6, P = 0.02) (Fig 8A). Correlations were also found between UCP-1 and Ang (1–7) (r = 0.6, P = 0.019) and Ang (1–7)/Ang II ratio (r = 0.7, P = 0.0037) (Fig 8B and 8C). The correlation between UCP-1 and Ang II was not different (Fig 8D).

## Discussion

The present study proposed to investigate the effects of AET on the thermogenic response, substrate metabolism and RAS in the WAT-SC of mice fed cafeteria diet. For this, we firstly tested the efficacy of the AET to promote aerobic adaptation through a maximal physical test after AET. According to the results it was observed the favorable adaptation to AET in both trained groups. This result corroborates previously studies published in the literature [3, 7, 50]. It is already known in the literature that the improvement in the physical performance of trained animals establishes a direct relation between the improvement of the aerobic capacity and reduction of the risk of cardiovascular and metabolic diseases [55, 56].

Despite some determinants of daily energy expenditure were not evaluated in this study and that the assessment of resting energy expenditure refers only to a period of 45 min, our results indicate that body weight response in trained mice could be due to the energy expenditure during AET since food intake and resting metabolic rate did not change. Furthermore, the respiratory exchange ratio and the rates of carbohydrates and lipids oxidation were not different among groups, revealing that neither AET nor diet induced substrate switch for oxidation or improved peripheral metabolic substrate utilization.

The food intake result corroborates our previous published paper with the same experimental protocol [3]. Considering that energy balance is determined by caloric intake and caloric expenditure, one possible explanation to the higher body weight and adiposity showed by CAF-SED is the metabolic activity of WAT induced by cafeteria diet, since caloric intake and expenditure were not different between CAF-SED and CHOW-SED. It is possible that cafeteria diet improves proteins and enzymes associated with lipogenesis and/or reduces proteins and enzymes associated with lipolysis. We previous observed that cafeteria diet damages the ability of visceral fat pads to handle excess lipids via alterations in enzymes responsible for fatty acid esterification [3]. As discussed previously [57], the effect of diet composition on body weight or body composition is not necessarily "a calorie is a calorie". Isocaloric diets that differ in macronutrient composition may result in preferential energy storage toward the adipose tissue. Diets with high protein content appears to offer advantages to over body composition and diets high in carbohydrates raise insulin secretion, favoring the uptake of glucose by the adipose tissue. On the other hand, fat does not stimulate insulin secretion. Thus, isocaloric diets with lower carbohydrate content and higher fat content reduce insulin secretion, making free fatty acid available for use by metabolically active tissues.

It is well established in the literature that AET promotes beneficial effects in parameters such as glycemia, lipid profile and body adiposity [3, 21, 58]. Our results showed that the cafeteria diet induced higher body weight gain, adiposity, glucose intolerance and IR in the CAF-SED group, and AET was able to prevent such damages in the CAF-TR group. The increase in WAT fat pads observed in this model contributes to the establishment of IR and glucose intolerance in these animals, due to the alteration in the secretion of adipokines leading to the damage in the insulin signaling pathway [59–62].

Glucose metabolic pathway was studied in the SC-WAT, and the reduction of the p-AKT/t-AKT ratio in both trained groups suggests that AET decreases insulin signaling, which can contribute to the reduction of *de novo* lipogenesis in trained groups [63]. On the other hand, CAF-SED group showed no difference in the p-AKT/t-AKT ratio suggesting a lower participation of this fat pad in the reduction of insulin sensitivity observed in CAF-SED. Furthermore, diet and AET did not modify the protein expression of the GLUT-4 transporter. Although the increase in GLUT-4 expression induced by AET in the skeletal muscle has already been demonstrated [64, 65] and that previous studies have observed a reduction of GLUT-4 in the WAT of animals with IR and T2D [66–69], in our experimental model such responses did not occur. It is important to note that the effect of diets and AET on the GLUT-4 translocation to the membrane could not be assessed, since our technique quantified the total GLUT-4 content in the cell. Thus, we cannot rule out the possibility of increased GLUT-4 content in the membrane.

In the lipid metabolic pathway, the CAF-TR group increased the t-HSL expression and the p-HSL / t-HSL ratio and AET was also able to increase ATGL protein expression in both trained groups compared with both sedentary groups. Considering that HSL and ATGL are essential for the hydrolysis of triglycerides [70,71], these results corroborate the weight of the SC-WAT fat pad in CAF-TR group, since AET prevented the increase of this fat pad induced by the cafeteria diet.

Thermogenesis / browning markers were evaluated by both gene and protein expression. The cafeteria diet reduced the gene expression of UCP-2 and CIDEA in the CAF-SED group, which may show a reduction in thermogenesis and energy expenditure in the SC-WAT fat pad. These results can explain the higher SC-WAT weight and adipocyte hypertrophy observed in CAF-SED. The increase in UCP-1 protein expression and more than 100% increase in UCP-1 and CIDEA gene expression in the CHOW-TR group reveals the potential of AET for the induction of browning in the SC-WAT. This finding has already been demonstrated previously by other authors [72,73]. Interestingly, the increase of browning marker in SC-WAT did not lead to lower SC-WAT weight, diameter or area of the adipocytes in CHOW-TR. It is possible that the lipolytic activity, and consequently the availability of fatty acid for oxidation, are a limiting step for SC-WAT thermogenesis, since the CHOW-TR group increased only ATGL protein expression.

Regarding the components of RAS in serum, ACE activity did not change among groups, but ACE2 activity increased in the CHOW-TR group, suggesting an association between AET and ACE2 activity modulation and that cafeteria diet precluded this response in the CAF-TR group. However, AET increased circulating Ang (1–7) concentration in both CHOW-TR and CAF-TR groups. Previous researches showed that the improvement of circulating Ang (1–7) induced by chronic systemic Ang (1–7) administration provided significant reduction in body weight and adipose tissue mass, decreased total cholesterol and triglycerides, increased insulin sensitivity, glucose tolerance, and decreased the expression of proinflammatory cytokines mRNA [38,74,75]. According to Loloi et al. [38], systemic Ang (1–7) produced direct insulin-sensitizing effects on skeletal muscle by reducing protein levels of AS160, a negative regulator of Glut4 translocation to the sarcolemma. Thus, the increase in serum Ang (1–7) level could probably counteract the deleterious effect of cafeteria diet because AET prevented obesity and IR in CAF-TR group when compared with CAF-SED group. One limitation of this study was the non-use of blood inhibitor cocktail to block renin and other peptidases that process or metabolize angiotensin peptides.

In the SC-WAT, the reduction of angiotensinogen in CHOW-TR may indicate less local production of the peptide or the activation of the RAS axis due to the higher peptide cleavage [31, 32]. As the Ang I concentration was not evaluated, and the Ang II results did not differ among groups, it is possible that the AET caused a reduction in the angiotensinogen expression in the CHOW-TR group.

Although there was no change in ACE activity and Ang II concentration in the SC-WAT, we observed an increase in the expression of AT1 and AT2 receptors in the CAF-TR group. Considering that Ang II acts preferentially through the AT1 receptor, it is possible that the CAF-TR has an increase in the action of Ang II due to the greater availability of the AT1 receptor. In this sense, the hyperactivity of the RAS axis through AT1 in the WAT can inhibit adipogenesis [29, 32], reduce lipolysis and browning [29, 31, 76]. This result may be associated with the browning marker data presented by CAF-TR, since the AET combined with the cafeteria diet precluded the increase of browning markers induced by AET. On the other hand, we cannot rule out the increase of AT2 expression in the CAF-TR group, since SC-WAT fat pad weight, adipocyte area and diameter were smaller in this group. Furthermore, although the expression of AT2 did not differ statistically in CHOW-TR, the increase of 50% could contribute to the greater action of Ang II via AT2. It is known that Ang II via the AT2 receptor presents opposite actions to those performed via the AT1 receptor. In fact, chronic treatment with the AT2 agonist (Compound 21) has been shown to reduce the size of hypertrophied adipocytes and WAT mass in rodents fed a high-fat diet, suggesting the favorable effects of AT2 activation for the treatment of obesity [77]. Further studies with receptor antagonists are necessary to clarify the activation of AT1 and AT2 receptors in our experimental model.

In the non-classic RAS axis, the cafeteria diet increased ACE2 activity in the CAF-SED and CAF-TR groups. In CHOW-TR, we observed higher Ang (1–7) levels even without increased ACE2 activity, suggesting that the increase of the peptide may be due to the action of other enzymes, such as prolil endopeptidase (PEP), prolyl carboxypeptidase (PCP) or neutral endopeptidase (NEP) [31]. The actions of Ang (1–7) through Mas receptor in the WAT include increase of adipogenesis [77], lipolysis and browning, reduction of lipogenesis and improvement in glucose and IR tolerance, which confirms our metabolic and browning results of WAT-SC in the CHOW-TR group [31, 32, 36]. In addition, it has been described the action of Ang (1–7) also via AT2 receptor, triggering functions like increase of adipogenesis, lipogenesis and activation of browning [77]. The increase in Ang (1–7) together with the higher Ang (1–7)/Ang II ration suggest an imbalance in the RAS towards the ACE2/Ang-(1–7)/Mas receptor axis and provide evidence that CHOW-TR animals may be more susceptible to the beneficial actions of Ang (1–7). Moreover, considering the increase in serum ACE2 and Ang (1–7) in CHOW-TR group, we can not exclude that local WAT RAS changes are dependent of the systemic RAS.

The concentration of Ang (1–7) and the Ang (1–7)/Ang II ration were not different in the CAF-TR group. Despite the increase in the expression of the Mas receptor, metabolic and browning response of SC-WAT were not associated with tissue Ang (1–7). Positive correlations observed between the expression of UCP-1 and the kITT (r = 0.6), between UCP-1 and Ang (1–7) concentration (r = 0. 6), and between UCP-1 and Ang (1–7)/Ang II (r = 0.7) suggest the association of browning with improvement in insulin sensitivity and higher concentration of Ang (1–7). These results were widely identified in the CHOW-TR group, however the protective effect of AET against obesity and IR was not associated with browning response and changes in the SC-WAT Ang (1–7) concentration of CAF-TR group.

## Conclusion

In conclusion, the AET prevented obesity and IR, reduced insulin signaling proteins and increased lipolysis signaling proteins in the SC-WAT. In addition, the CAF diet precludes the AET-induced thermogenic response and the partial modulation of the RAS suggests that the protective effect of AET against obesity and IR could not be associated with SC-WAT RAS.

## Supporting information

S1 Fig Physical performance before AET (A), physical performance after AET (B). CHOW-SED (n = 10), CHOW-TR (n = 10), CAF-SED (n = 10) and CAF-TR (n = 10). Error bars indicate the SE. p≤ 0.05 *CHOW-TF vs. CHOW-SED, CAF-SED and CAF-TR; ^#^CAF-TR vs. CAF-SED.
(TIFF)

S1 Table Fasting glucose, área under the curve (AUC) and rate constant for the disappearance of plasma glucose (kITT). Data are presented as mean ± SE. *p ≤ 0.05 vs. CAF-SED; ^#^p ≤ 0.05 vs. CHOW-SED, CHOW-TR and CAF-TR.
(DOCX)
