# Supplementary figures and images for: Aerobic exercise training prevents obesity and insulin resistance independent of the renin angiotensin system modulation in the subcutaneous white adipose tissue
Source: PLoS One. 2019 Apr 25;14(4):e0215896. doi: 10.1371/journal.pone.0215896 (PMC6483229; doi:10.1371/journal.pone.0215896)

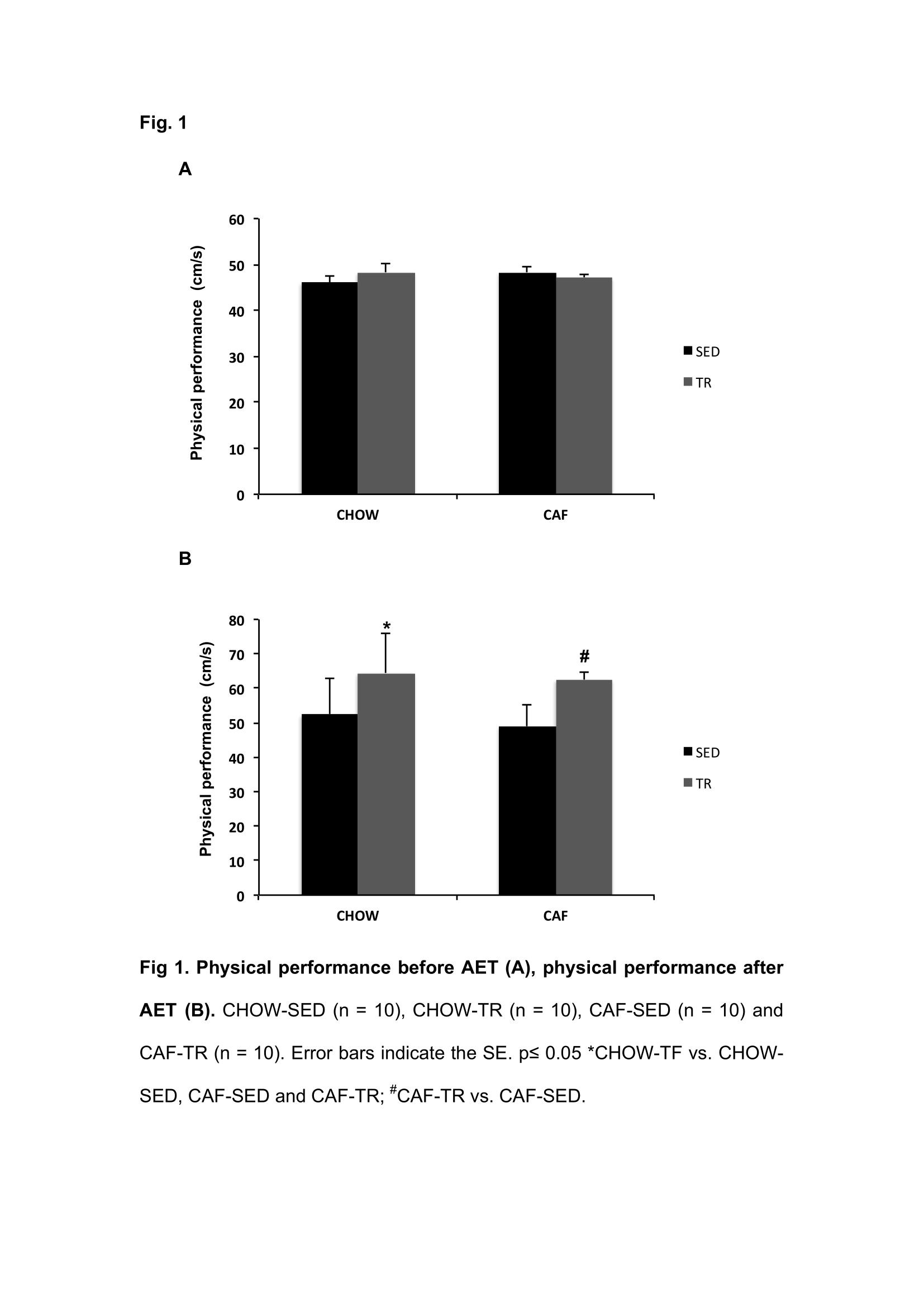

Supplement: S1 Fig — CHOW-SED (n = 10), CHOW-TR (n = 10), CAF-SED (n = 10) and CAF-TR (n = 10). Error bars indicate the SE. p≤ 0.05 *CHOW-TF vs. CHOW-SED, CAF-SED and CAF-TR; #CAF-TR vs. CAF-SED. (TIFF) [file pone.0215896.s001.tiff]
