# Supplementary material for: Aerobic exercise training prevents obesity and insulin resistance independent of the renin angiotensin system modulation in the subcutaneous white adipose tissue
Source: PLoS One. 2019 Apr 25;14(4):e0215896. doi: 10.1371/journal.pone.0215896 (PMC6483229; doi:10.1371/journal.pone.0215896)
Supplement: S1 Table — Data are presented as mean ± SE. *p ≤ 0.05 vs. CAF-SED; #p ≤ 0.05 vs. CHOW-SED, CHOW-TR and CAF-TR. (DOCX) [file pone.0215896.s002.docx]

**Table 1: Fasting glucose, área under the curve (AUC) and rate constant for the disappearance of plasma glucose (kITT).**

|  | CHOW-SED  (n=10) | CHOW-TR  (n=10) | CAF-SED  (n=10) | CAF-TR  (n=10) |
| --- | --- | --- | --- | --- |
| Fasting Glucose (mg/dL) | 123 ± 4 | 114 ± 2* | 133 ± 7 | 129 ± 6 |
| AUC (mg/dL/120min) | 29156 ± 825 | 24777 ± 1387 | 37505 ± 1601^#^ | 28423 ± 1310 |
| kITT  (%/min) | 3.72 ± 0.21 | 4.09± 0.36 | 2.5 ± 0.17^#^ | 3.94 ± 0.33 |

Data are presented as mean ± SE. *p ≤ 0.05 vs. CAF-SED; ^#^p ≤ 0.05 vs. CHOW-SED, CHOW-TR and CAF-TR.
